# Chromosomal-level genome assembly and annotation of the tropical sea cucumber *Holothuria scabra*

**DOI:** 10.1038/s41597-024-03340-x

**Published:** 2024-05-09

**Authors:** Shengping Zhong, Xujia Liu, Xiaowan Ma, Xiuli Chen, Yan Jiang, Mengqing Zeng, Longyan Zhao, Lianghua Huang, Guoqiang Huang, Yongzhen Zhao, Hongtao Liu, Ying Qiao

**Affiliations:** 1 Guangxi Key Laboratory of Marine Drugs, Institute of marine drugs, Guangxi University of Chinese Medicine, Nanning, 530200 China; 2 Guangxi Engineering Technology Research Center for Marine Aquaculture, Guangxi Institute of Oceanology Co., Ltd., Beihai, 536000 China; 3 Guangxi Key Laboratory of Marine Environmental Science, Guangxi Academy of Marine Sciences, Guangxi Academy of Sciences, Nanning, 530007 China; 4 Key Laboratory of Tropical Marine Ecosystem and Bioresource, Fourth Institute of Oceanography, Ministry of Natural Resources, Beihai, 536000 China; 5 Guangxi Key Laboratory of Aquatic Genetic Breeding and Healthy Aquaculture, Guangxi Academy of Fishery Sciences, Nanning, 530007 China; 6 Hainan Provincial Key Laboratory of Tropical Maricultural Technologies, Hainan Academy of Ocean and Fisheries Sciences, Haikou, 570100 China

**Keywords:** Marine biology, Genomics, Molecular ecology, Ecological genetics, Ecological genetics

## Abstract

*Holothuria scabra*, a commercially valuable yet ecologically vulnerable tropical holothuroid, has experienced a severe decline in its wild populations, especially in China. Genomic resources are crucial for the development of effective genomic breeding projects and stock conservation strategies to restore these natural populations. Until now, a high-quality, chromosome-level reference genome for *H. scabra* has not been available. Here, we employed Oxford Nanopore and Hi-C sequencing technologies to assemble and annotate a high-quality, chromosome-level reference genome of *H. scabra*. The final genome comprised 31 scaffolds with a total length of 1.19 Gb and a scaffold N50 length of 53.52 Mb. Remarkably, 1,191.67 Mb (99.95%) of the sequences were anchored to 23 pseudo-chromosomes, with the longest one spanning 79.75 Mb. A total of 34,418 protein-coding genes were annotated in the final genome, with BUSCO analysis revealing 98.01% coverage of metazoa_odb10 genes, marking a significant improvement compared to the previous report. These chromosome-level sequences and annotations will provide an essential genomic basis for further investigation into molecular breeding and conservation management of *H. scabra*.

## Background & Summary

Echinoderms are a group of deuterostome invertebrate animals^1^, comprising two other closely related phylogenetic groups: hemichordates and chordates^2^. Echinoderms are the largest marine phylum among deuterostome animals, with more than 7,000 living species^3^. They are found exclusively in marine environments and are known to inhabit diverse marine environments, including shallower oceans, the deep oceans, as well as intertidal zones^4^. Sea cucumbers, or holothuroids (Echinodermata: Holothuroidea), are a species-diverse echinoderm group with significant ecological and economic roles in marine ecosystems as important benthic invertebrates^5^. There are more than 1,700 confirmed holothuroid species in the world, with the greatest diversity located in the Asia-Pacific region^6^. Nevertheless, there are fewer than 100 holothuroid species are considered suitable for consumption and are currently being commercially harvested worldwide^7^. Among them, only a few species such as *Apostichopus japonicus* have undergone artificial breeding techniques and are being cultivated as a delicious seafood^8^. In recent years, increasing consumer demand in the Asian region has caused severe overfishing of numerous commercially valuable holothuroid species, leading to a significant depletion of their natural populations. The development of artificial breeding techniques and effective fishery management of sea cucumbers needs urgent investigation.

*Holothuria scabra*, or sandfish, is among the commercially valuable yet ecologically vulnerable tropical holothuroids^9^. Because of its rich flavor and great nutritional value, *H. scabra* is considered one of the most precious tonic foods with significant commercial value in the Asian market^10^. Since the 1970s, when commercial harvests increased dramatically, *H. scabra* has been intensively exploited in the Pacific and Indian oceans, including China, Indonesia, India, Philippines, and Australia^11^. In recent decades, a growing demand in the Asian market, coupled with insufficient fishery management of *H. scabra*, has led to the depletion of the natural stocks across its entire geographic range, particularly in China^9^. The wild populations of *H. scabra* have not been observed in the Beibu Gulf of China for more than a decade now, despite the fact that natural stocks of this species were once abundant in this region during the 1960s^12^. Owing to the dramatic decline in the wild stocks of *H. scabra*, the International Union for Conservation of Nature (IUCN) designated *H. scabra* as an endangered species in 2013^13^. In order to recover *H. scabra*’s severely depleted natural populations, it is imperative to develop effective aquaculture techniques and population enhancement projects of *H. scabra*. However, thus far, artificial breeding techniques for *H. scabra* have been applied inefficiently in China, and the scale of aquaculture for *H. scabra* has been limited^14^.

In recent times, high-quality chromosome-level reference genomes of commercially valuable holothuroid species, such as *A. japonicus*^8,15^ and *Holothuria leucospilota*^16^, have been published and are now readily accessible to researchers. These genomic resources are essential for implementing more efficient genomic breeding projects and stock conservation strategies aimed at restoring natural populations. However, thus far, high-quality chromosome-level reference genome of *H. scabra* has not been published. Even though *H. scabra*’s genomic assembly and annotation were reported in 2022, the genome sequences and annotation data are still regrettably inaccessible to researchers. In this report, using multiple sequencing technologies, a high-quality chromosome-level reference genome of *H. scabra* was constructed and annotated. Approximately 1.19 Gb of genomic sequences were assembled into a chromosome-level genome, consisting of 31 scaffolds, with a scaffold N50 length of 53.52 Mb and a total of 528 gaps. Specifically, 1,191.67 Mb (99.95%) of the sequences were anchored to 23 pseudo-chromosomes, with the longest one spanning 79.75 Mb. In total, 34,418 protein-coding genes were annotated, and the BUSCO analysis demonstrated coverage of 98.01% of metazoa_odb10 genes, marking a significant improvement compared to the previous report. The availability of the first chromosome-level genome sequences and annotations for *H. scabra* represents a valuable genomic resource. It will play a pivotal role in enabling more efficient genomic breeding projects and stock conservation strategies, which are essential for restoring the severely depleted stocks of *H. scabra* in China.

## Methods

### Sample collection and sequencing

A healthy male sandfish with a body weight of 82.30 g was collected from Hainan Province, China, (coordinates: 19.25 N, 110.64 E) for genomic DNA sequencing, transcriptome sequencing, and chromosome conformation capture (Hi-C) sequencing. To perform genomic DNA sequencing, we extracted high molecular weight (HMW) genomic DNA from a muscle sample using the QIAamp DNA Mini Kit (QIAGEN, Hilden, Germany). Subsequently, we utilized a combination of Nanopore and MGI platforms to obtain the genomic sequences of *H. scabra*. Approximately 46.10 Gb of long read sequences averaging 18,500 bp in length were obtained from the Nanopore 20 kb insert sequencing library. Meanwhile, the MGI 350 bp insert sequencing library yielded 113.56 Gb of paired-end sequences with 2 × 150 bp lengths and Q20 > 98.58%. To conduct transcriptome sequencing, total RNA was extracted from various tissue samples, including tentacles, respiratory tree, and intestine, using the RNAiso kit (TaKaRa, Tokyo, Japan). We utilized the MGISEQ-2000 platform to generate approximately 74.76 Gb of transcriptome data with a Q20 quality score of 98.64%. In order to perform Hi-C sequencing, a sample of freshly harvested muscle was first formaldehyde cross-linked and then digested using the DpnII restriction enzyme. By using the Illumina NovaSeq platform, approximately 168.60 Gb of clean paired-end reads with a Unique Mapped Ratio of 60.45% were generated from the Hi-C sequencing library (Table 1).

**Table 1:** Summary of obtained sequencing data generated for *H. Scabra* genome assembly and gene prediction.

Sequencing libraries	Insert size	Clean data (Gb)	Read Length (bp)	Coverage (X)
Nanopore	20 k	46.10	18,500(Average)	39.07
BGISEQ-500	350 bp	113.56	150	96.24
RNA-seq	—	74.76	150	63.36
Hi-C	300–700	168.60	150	142.88
Total	—	406.93	—	341.54

### Genome survey and assembly

**Fig. 1:**
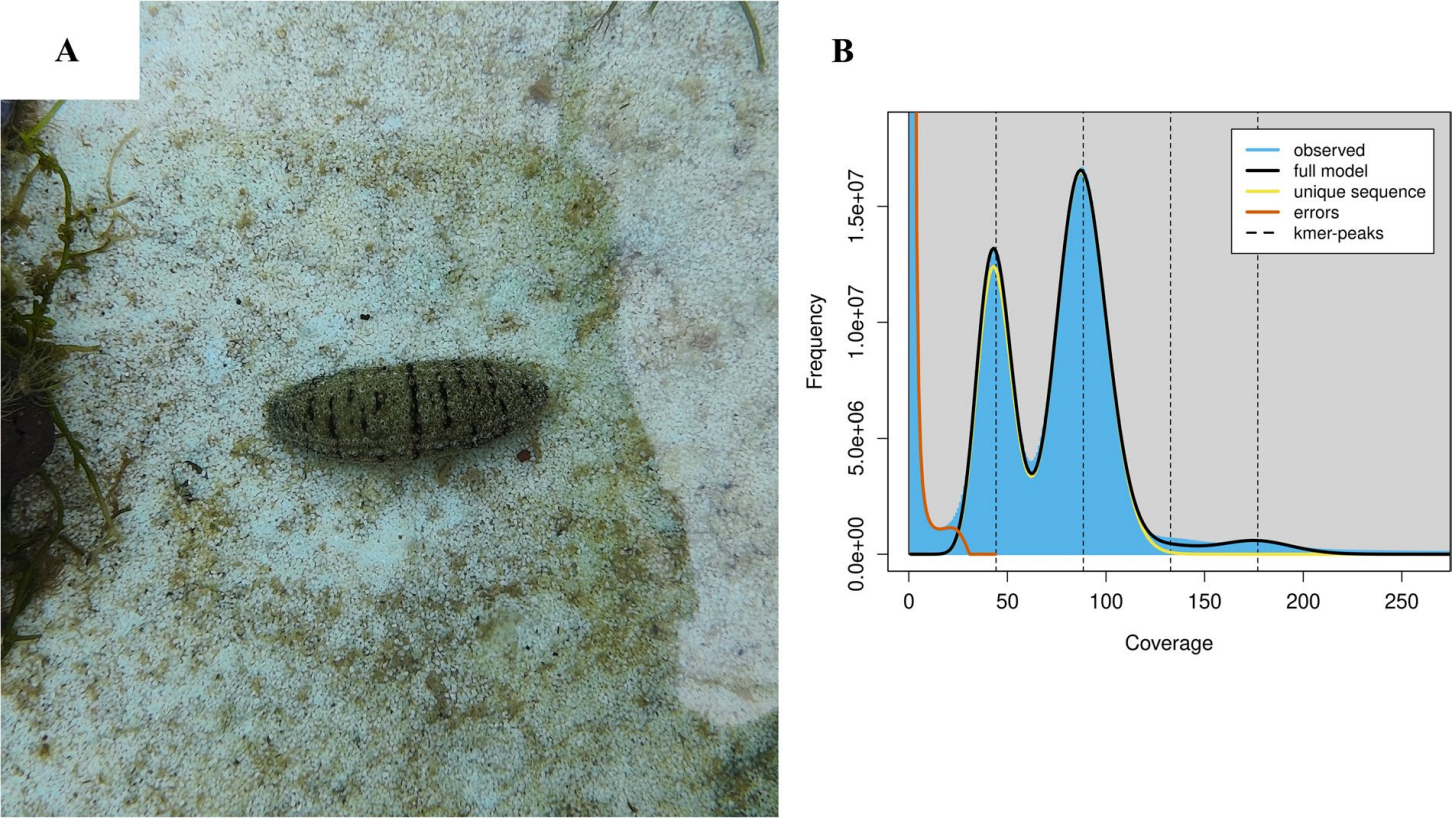
*H. scabra* and the genomics feature. (**A**) *H. scabra* reared in outdoor pond. (**B**) A K-mer analysis of *H. scabra* genomics feature.

**Fig. 2:**
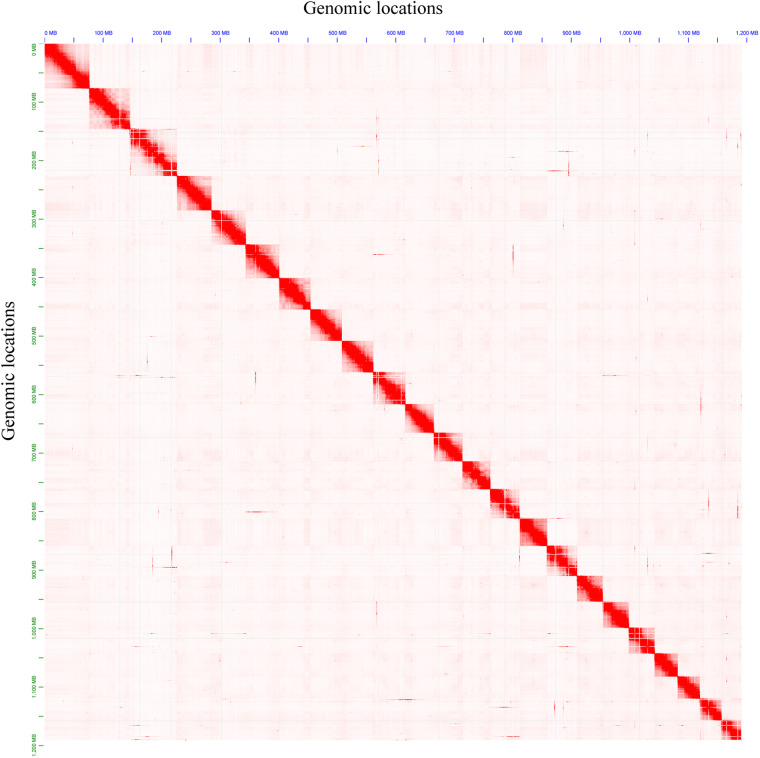
The Hi-C interaction map of final chromosomal-level genome of *H. scabra*. The color demonstrates the intensity of the interaction from white (low) to red (high).

**Fig. 3:**
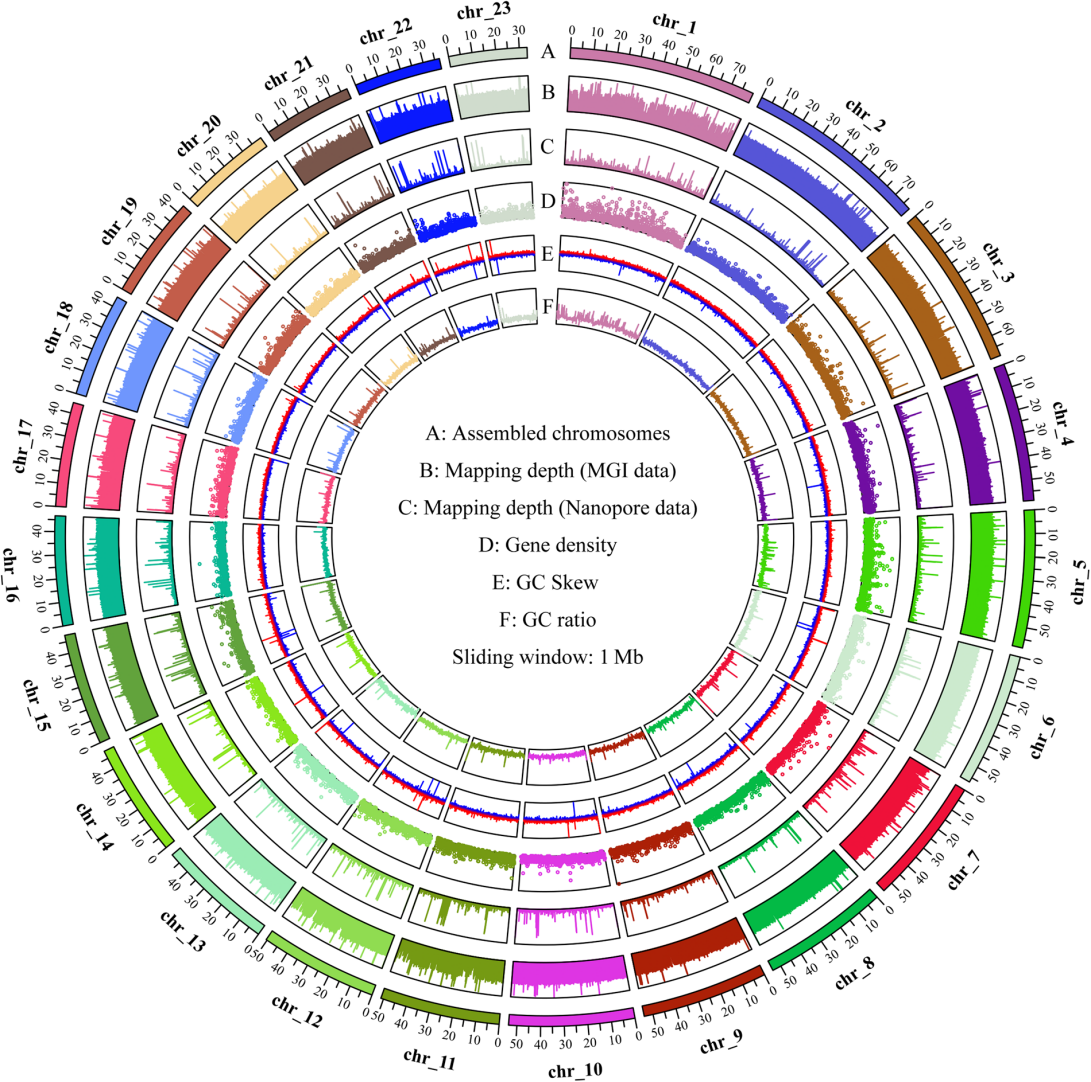
Genomic landscape of the 23 assembled chromosomes of *H. scabra*. Sliding window: 1 Mb; (**A**) Assembled chromosomes; (**B**) Mapping depth (MGI data); (**C**) Mapping depth (Nanopore data); (**D**) Gene density; (**E**) GC Skew; (**F**) GC ratio.

The haploid genome length and polymorphism information of *H. scabra* were assessed through k-mer analysis using the genomic paired-end reads from the MGI sequencing platform. Initially, k-mer frequencies with a length of 19 were calculated using Jellyfish (v.2.3.0)^17^. Subsequently, Genomescope (v.2.0)^18^ was employed to calculate genomic characteristics for *H. scabra* based on the frequency information. The results revealed a predicted haploid genome size of 1127.23 Mb, with repetitive rate and heterozygous rate of 41.73% and 1.24%, respectively (Fig. 1). In order to assemble the high heterozygosity regions of *H. scabra* genome, the long read data from Nanopore sequencing platform were used by NextDenovo (v.2.5.2) with a correct-then-assemble strategy. Subsequently, the high accuracy genomic paired-end reads data were used by NextPolish (v.1.4.1)^19^ to improve the base accuracy of contigs. Finally, the redundancy regions in corrected contigs were eliminated by Purge_Dups (v.1.2.6)^20^. Eventually, we assembled a contig-level genome for *H. scabra*, consisting of 505 contigs with a total size of 1192.13 Mb, a contig N50 length of 3.15 Mb, and the longest contig spanning 19.72 Mb. In order to anchor the contig sequences to chromosomes, a Hi-C scaffolding tool called YaHS (v.1.1)^21^ was applied for mapping all Hi-C paired-end reads to the contig-level genome of *H. scabra* with default parameters. For correction and refinement of the draft scaffold genome, Juicebox (v.3.1.4)^22^ was used for manual reviewing and Hi-C interaction map generation. The Hi-C interaction map of the final chromosomal-level genome showed a clear interaction signal of 23 super-scaffolds indicating that the number of pseudo-chromosomes in *H. scabra*’s genome was 23 (Fig. 2). The result of the genome-wide interaction signal of *H. scabra* was consistent with the previous investigation in the *holothuria* species^16^ and *A. japonicus*^8^. The final chromosomal-level genome had a size of 1.19 Gb and consisted of 31 scaffolds, with a scaffold N50 length of 53.52 Mb and gap length (N’s) of 52,800. Of the final genomic sequences, 1,191.67 Mb (99.95%) of the sequences were anchored to 23 pseudo-chromosomes, with lengths ranging from 79.75 Mb to 33.31 Mb (Table 2 & Fig. 3), and contained 525 gaps. To evaluate the functional completeness of the final chromosomal-level genome, we conducted a BUSCO (v.5.4.4)^23^ analysis in genome mode using the metazoa_odb10 dataset. The results indicated that it covered 97.90% of metazoa_odb10 genes, comprising a completion rate of 94.55% and a fragmentation rate of 3.35%, with only a missing rate of 2.10% (Table 2). Compared to the previous genome assembly report of *H. scabra*, our chromosomal-level assembled genome has shown significant improvements in terms of genomic contiguity and completeness (Table 2).

**Table 2:** Comparative statistic of genome assembly and gene prediction of *H. Scabra* with previous report.

Summary statistics of contig level genome assembly	this study	Luo *et al.*
Total length of genome (Mbp)	1,192.13	1,181.45
Contig N50 size (Mbp)	5.30	1.56
Contig N90 size (Mbp)	1.34	0.13
Contig number	505	4372
The length of largest contig (Mbp)	19.72	11.13
Proportion of BUSCO in genome model (%)	97.90	91.11
**Summary statistics of scaffold level genome assembly**		
Total length of genome (Mbp)	1192.18	—
Scaffold N50 size (Mbp)	53.52	—
Scaffold N90 size (Mbp)	39.54	—
Scaffold number	31	—
The length of largest Scaffold (Mbp)	79.75	—
Proportion of BUSCO in genome model (%)	97.90	—
**Summary statistics of gene prediction**		
Protein-coding gene number	34,418	16,642
The length of largest protein-coding gene (bp)	53,703	—
Mean gene length (bp)	16,859	25,967
Mean exon length (bp)	208	—
Mean exons number per gene	5.70	—
Proportion of BUSCO in proteins model (%)	98.74	—

### Transposable elements and noncoding RNAs (ncRNAs) annotation

To evaluate the presence of transposable elements (TE) in the genome of *H. scabra*, a combined approach utilizing *ab initio* and homology-based strategies was employed by EDTA (v.2.1.0)^24^ and RepeatMasker (v.4.1.2, www.repeatmasker.org), respectively. The TE of *H. scabra*’s genome was first predicted with *ab initio* strategy, and then based on the predicted TE library, homology-based strategy was used to find out the remaining TE sequences. The final TE prediction analysis revealed that 52.31% of the *H. scabra* genome, equivalent to a total length of 623.64 Mb, consisted of transposable elements, slightly higher proportion compared to *H. leucospilota* (50.41%). Specifically, the *H. scabra* genome exhibited the most abundant of terminal inverted repeats, accounting for 35.81% of the genomic sequences. Long terminal type and tandem type were the following abundant types, accounting for 8.84% and 4.43%, respectively (Table 3). In order to predict the ncRNAs in the genomic sequence, tRNAscan-SE (v.2.0.6)^25^ and RNAmmer (v.1.2)^26^ were first applied to search transfer RNAs (tRNAs) and ribosomal RNAs (rRNAs). Pre-miRNAs and other remaining ncRNAs were searched by Infernal (v.1.1.2)^27^ based on the Rfam datasets. A set of 0.97 Mb genomic sequences was predicted to be ncRNAs including 5,836 tRNAs, 1,978 pre-miRNAs, 99 rRNAs, and 1699 snRNAs (Table 4).

**Table 3:** Summary statistics for the annotated repeat sequences.

Repeat Classes	Count	Length (bp)	% in genome
terminal inverted repeats	1,374,186	426,823,371	35.81
long terminal repeats	282,416	105,407,467	8.84
non-long terminal repeats	18,641	8,376,228	0.70
helitron	119,110	30,182,221	2.53
tandem repeats	152,095	52,853,142	4.43
Total	1,946,448	623,642,429	52.31

**Table 4:** Summary statistics of non-coding RNA annotation.

	Type	Number	Average Length(bp)	Total Length(bp)	% in Genome
pre-miRNAs	—	1,978	90	178,445	0.02
tRNAs	—	5,836	72	420,192	0.04
rRNAs	5 S	77	121	9,184	0.00
	18 S	8	1512	10,583	0.00
	28 S	14	2406	31,282	0.00
	Total	99	4039	51,049	0.00
snRNAs	CD-box	19	106	1,906	0.00
	HACA-box	7	185	1,107	0.00
	splicing	1699	189	321,188	0.03
	Total	1725	148	324,201	0.03

### Gene prediction and functional annotation

For the prediction of protein-coding genes, a combination of transcript-based, homology-based, and *ab initio* prediction methods was employed. Initially, transcriptome sequencing data from our study were employed to *de novo* assemble transcripts using Trinity (v.2.14)^28^ with default parameters. Subsequently, StringTie (v.2.2.1)^29^ was utilized to construct genome-guided transcripts. The predicted genes based on transcripts were then obtained by applying PASA (v.2.5.2)^30^ to map both the *de novo* and genome-guided transcripts. The validation of the homology-based method was conducted using GeMoMa (v.1.9)^31^ with default settings, relying on echinoderm protein data from GenBank, which included *Lytechinus pictus*^32^, *Anneissia japonica*^33^, *H. leucospilota*^34^, and *A. japonicus*^35^. For the *ab initio* approach, BRAKER (v.2.1.6)^36^ in combination mode was applied for predicting coding genes based on both the transcriptome sequencing data from this study and echinoderm protein data. Subsequently, the predictions from all three methods underwent evaluation using EvidenceModeler (v.2.1.0)^37^, followed by functional annotation with DIAMOND (v.2.1.3)^38^ and HMMER (v.3.4, hmmer.org). Default parameters were employed for searching the Swiss-Prot, UniProtKB-TremBL, Gene Ontology (GO), and KEGG databases, with an E-value limit of 1e-5 for homologous annotation. The completeness validation of the final predicted protein-coding genes was conducted through BUSCO (v.5.4.4) with the metazoa_odb10 datasets. The prediction of the *H. scabra* genome yielded a total of 34,418 protein-coding genes, with an average length of 16,859 bp (Table 2). According to BUSCO evaluation, these predicted genes covered 98.74% of metazoa_odb10 genes, with 98.01% complete genes (Table 5). Importantly, the completeness of gene predictions in the *H. scabra* genome exhibited a substantial improvement compared to the previous report (98.01% vs. 90.11%). Moreover, functional annotation was accomplished for 84.77% (Table 6) of *H. scabra* predicted genes across diverse protein databases, including UniProtKB-TremBL (83.30%), Swiss-Prot (55.17%), KEGG (60.13%), and GO (52.50%). Interestingly, the annotation ratios exhibited a notable similarity to those observed in *H. leucospilota*^16^, specifically in Swiss-Prot (55.80%), KEGG (66.11%), and GO (53.56%).

**Table 5:** The BUSCO result of *H. Scabra* genome chromosomal-level assembly and gene prediction.

	Term	Number	Ratio (%)
chromosomal-level assembly	Complete BUSCOS (C)	902	94.55
	Single-copy BUSCOS (S)	899	94.22
	Duplicated BUSCOS (D)	3	0.31
	Fragmented BUSCOS (F)	32	3.35
	Missing BUSCOS (M)	20	2.10
gene prediction	Complete BUSCOS (C)	935	98.01
	Single-copy BUSCOS (S)	927	97.17
	Duplicated BUSCOS (D)	8	0.84
	Fragmented BUSCOS (F)	7	0.73
	Missing BUSCOS (M)	12	1.26

**Table 6:** Statistics for the functional annotation of protein-coding genes.

Database	Gene Number	Percent (%)
Swiss-Prot	18,990	55.17
TremBL	28,670	83.30
GO	18,068	52.50
KEGG	20,697	60.13
At least one database	29,177	84.77
Total	34,418	—

## Data Records

The *H. scabra* genome assembly and annotation projects have been registered in the NCBI BioProject database under PRJNA1047316. The genomic sequencing data from both Nanopore and MGI platforms have been deposited in the NCBI Sequence Read Archive (SRA), with accession numbers SRR27010838^39^ and SRR27010031^40^, respectively. The transcriptome and Hi-C sequencing data are also stored in the NCBI SRA, with accession numbers SRR27022669^41^ and SRR27030181-SRR27030183^42^, respectively. The chromosomal-level genome assembly has been deposited in the NCBI GenBank with accession number GCA_037179385.1^43^. The chromosomal-level genome assembly and annotation files can be accessed through the Figshare^44^.

## Technical Validation

### DNA and RNA quality validation

Quality validation of genomic DNA samples using both Nanodrop spectrophotometer (LabTech, USA) and pulse electrophoresis in agarose gel. DNA samples with slightly degraded were considered viable for sequencing library construction. For RNA samples, quality was verified using an Agilent 2100 bioanalyzer (Agilent Technologies), with samples having an RNA integrity number (RIN) greater than 9.50 being considered suitable for library construction.

### Genome assembly and annotation quality evaluation

Quality validation of the genome assembly was initially conducted using QUAST (v.5.2 https://github.com/ablab/quast), which revealed a significant improvement in genome continuity for the final chromosome-level genome. In comparison with previous studies, the scaffold N50 of the *H. scabra* genome has increased markedly from 1.56 Mb to 53.51 Mb, and the length of the largest scaffold has grown from 11.12 Mb to 79.75 Mb (Fig. 3). Furthermore, the BUSCO completion score, evaluated using the metazoa_odb10 datasets, improved from 89.13% to 94.55% (Table 5). Merqury (v.1.3)^45^ was subsequently employed to evaluate the accuracy and completeness of the genome. The resulting consensus quality value (QV) of 53.37 and k-mer completeness of 91.31% suggest that the final chromosome-level genome assembly achieves a high degree of quality. Genomic sequencing data from Nanopore and MGI platforms were aligned using BWA (v.0.7.17)^46^ and minimap2 (v.2.28)^47^, respectively, to further validate the quality of the final genome assembly. The mapping rates for Nanopore and MGI sequencing data were 99.44% and 99.73%, respectively, while genome coverage rates were 99.99% and 99.65% (Table 8 & Fig. 3). These results indicate a high degree of quality in the final genome assembly. Lastly, the quality of the genome annotation was evaluated using the BUSCO (v5.4.4) software, based on the metazoa_odb10 datasets. This assessment revealed that the final genome annotation encompassed 98.74% of the metazoa_odb10 genes, demonstrating a high completeness rate in gene predictions. Additionally, we aligned transcriptome sequencing data using STAR (v.2.7.11a)^48^ software, set to spliced transcripts mode. This alignment process resulted in 91.01% of the sequencing reads being accurately mapped to the predicted genes, thereby confirming the high accuracy of our gene predictions.

Orthologous gene prediction and functional annotation evaluation

**Fig. 4:**
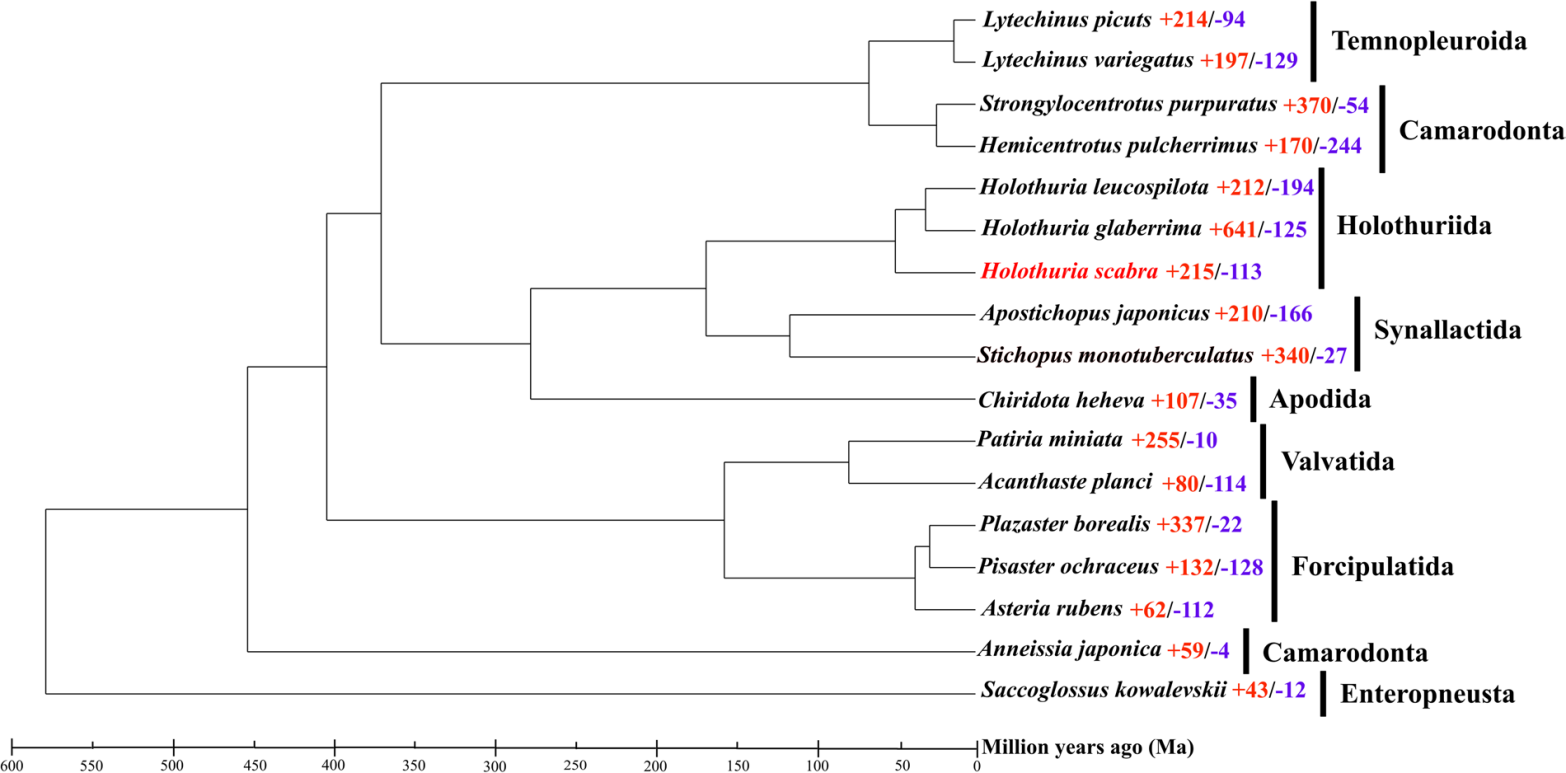
Comparative phylogenomics and gene family evolution of *H. scabra* and other species. The number of significantly expanded (red) and contracted (blue) gene families is designated beside species scientific name.

**Fig. 5:**
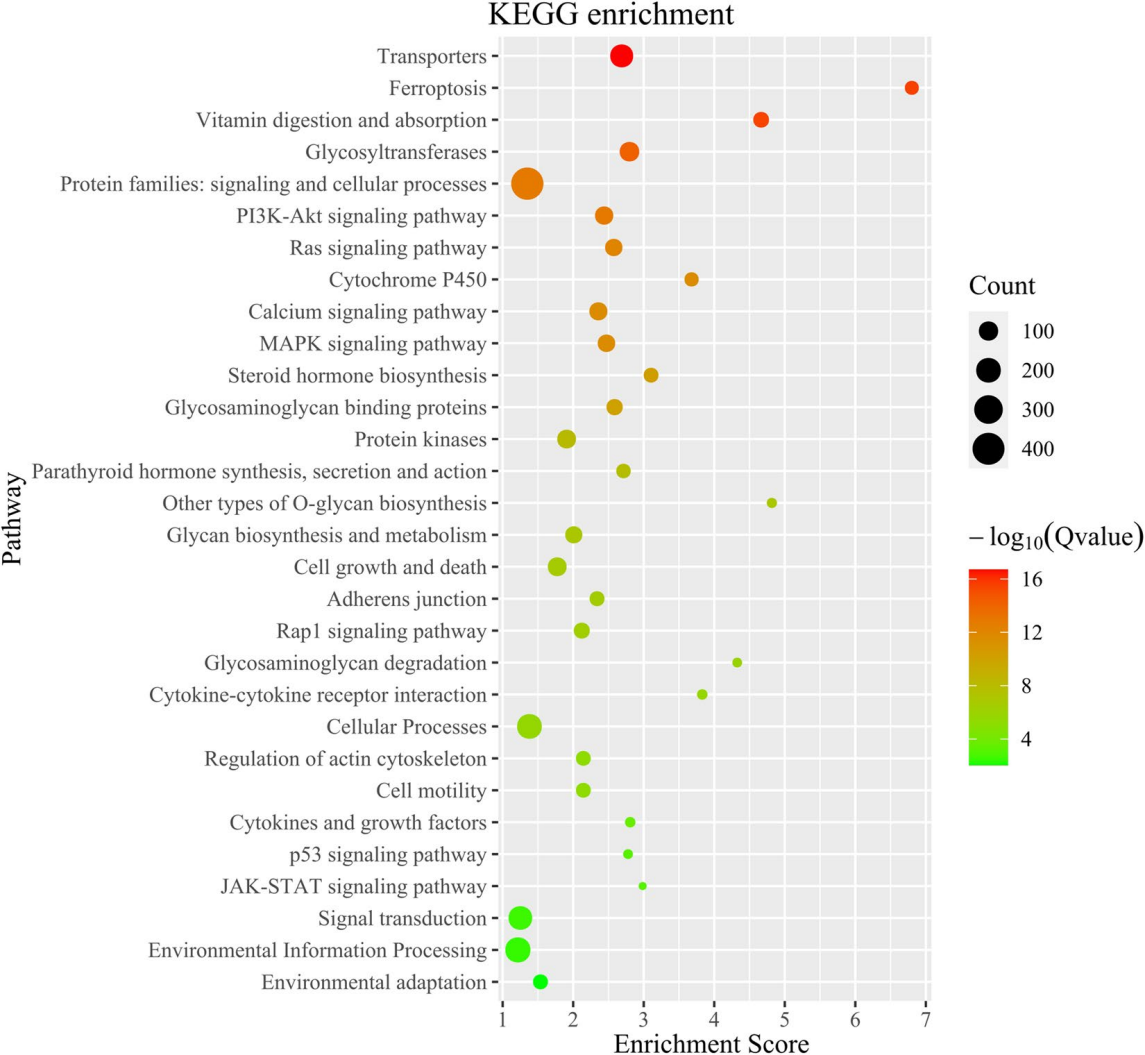
Kyoto Encyclopedia of Genes and Genomes (KEGG) enrichment analysis for significantly expanded gene families in *H. scabra* genome. Only the top 30 categories are shown.

**Fig. 6:**
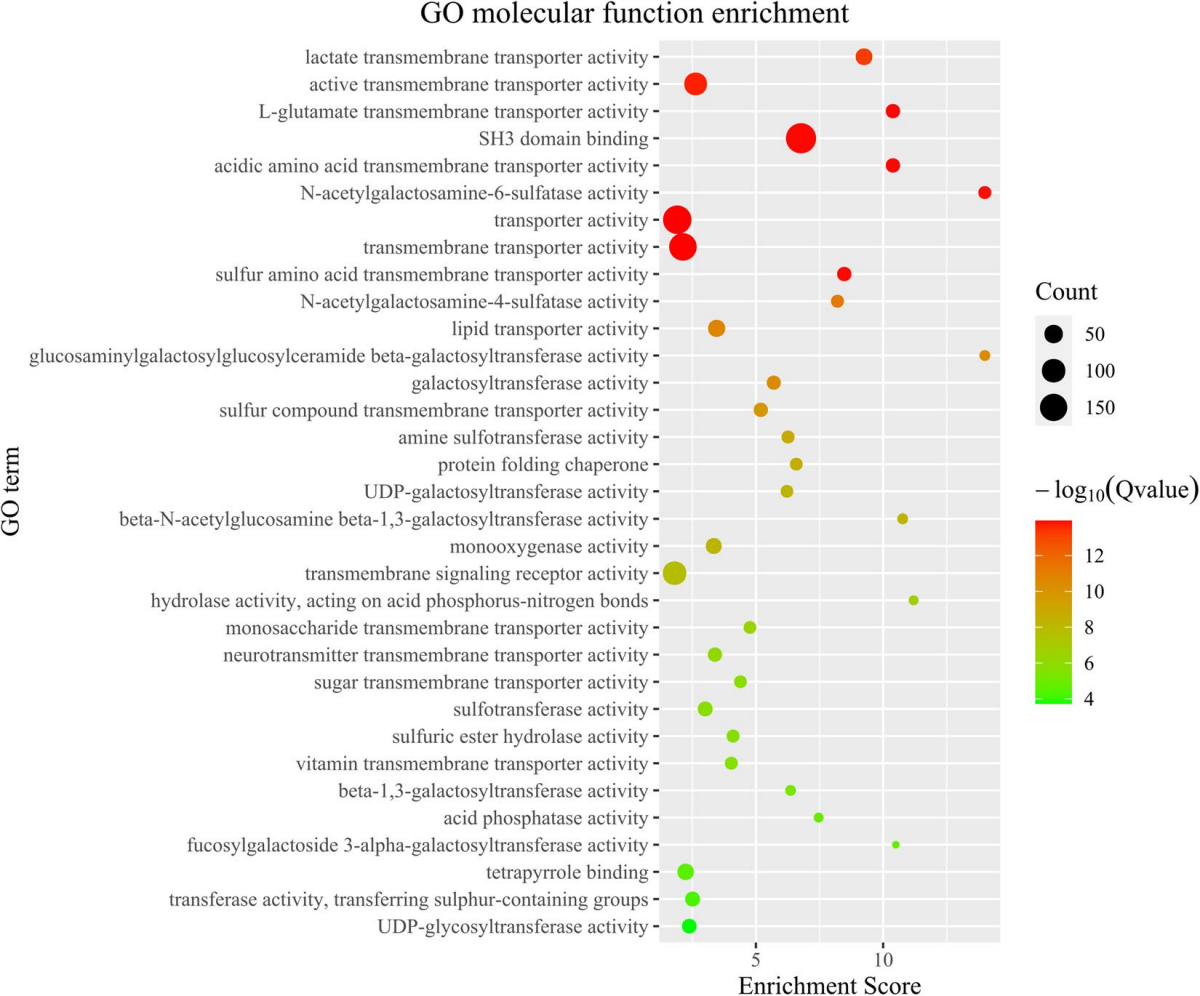
Function enrichment of Gene Ontology (GO) for significantly expanded gene families in *H. scabra* genome. Only the top 30 categories are shown.

The investigation into the orthologous gene clusters of *H. scabra* was conducted through OrthoFinder (v.2.5.4)^49^, utilizing genome-wide protein data from 16 related species obtained from GenBank and Figshare (Supplementary Table 1). Within the protein-coding genes of *H. scabra*, a total of 29,090 genes (84.51%) were identified as orthologous gene clusters, including 1,264 genes (3.71%) classified as species-specific type (Table 7). Additionally, the entire Echinodermata phylum shares 5,784 orthogroups, inclusive of 242 single-copy orthogroups. Sequence alignment of these single-copy orthogroups was conducted using MUSCLE (v.3.8.31)^50^, followed by the construction of a phylogenetic tree with RAxML (v.8.2.9)^51^, based on the super protein sequences integrated from these alignments, using the LG4M model and 1,000 bootstrap replicates. The phylogenomics analysis reveals a close relationship between *H. leucospilota* and *H. glaberrima* within the Holothuriidae clade, with *H. scabra* diverging from their shared ancestor at a later stage. Compared to the Apodida, the Stichopodidae and Holothuriidae families show a closer evolutionary relationship in the Holothuroidea clade. The evolutionary histories of gene families in *H. scabra* and 16 related species were examined using recalibrated evolutionary times for *Stichopus monotuberculatus* and *A. japonicus* obtained from TimeTree (www.timetree.org), processed with r8s (v.1.71) software. This was followed by a likelihood analysis comparing *H. scabra* to other Echinodermata species using the CAFÉ (v.5.0)^52^ tool with standard parameters. The likelihood analysis revealed that within the *H. scabra* genome, 359 gene families are unique, 215 are significant expansion, and only 113 are significant contraction, as illustrated in Fig. 4. Additionally, TBtools-II (v.2.0.81)^53^ was utilized for functional enrichment analyses to explore the biological functions of expanded gene families. The analyses revealed that these evolutionary gene families predominantly participate in biological processes related to signaling molecules and interaction, cytochrome P450, glycan biosynthesis and metabolism, glycosyltransferases, and environmental adaptation (Figs. 5, 6).

**Table 7:** Summary statistics for orthogroups in *H. Scabra* genome.

	*H. Scabra*
Number of genes	34418
Number of genes in orthogroups	29090
Number of unassigned genes	5328
Percentage of genes in orthogroups (%)	84.51
Percentage of unassigned genes (%)	15.48
Number of orthogroups containing species	14951
Percentage of orthogroups containing species (%)	46.92
Number of species-specific orthogroups	359
Number of genes in species-specific orthogroups	1264
Percentage of genes in species-specific orthogroups (%)	3.71

**Table 8:** Statistics of genomic DNA sequencing data mapped to *H. Scabra* genome.

sequencing platform	Mapping rate (%)	Average sequencing depth	Coverage (%)	Coverage (> = 10X,%)	Coverage (> = 30X,%)
Nanopore	99.44	66.81	99.99	99.86	98.64
MGI	99.73	91.44	99.65	99.06	97.06

### Supplementary information

Supplementary table 1.

## Data Availability

No custom scripts were utilized in this study. All commands and pipelines for data processing were carried out in compliance with the established protocols of the bioinformatics software, on a local high-performance server (PowerEdge T630, Dell Technologies).
