# Supplementary material for: Chromosomal-level genome assembly and annotation of the tropical sea cucumber Holothuria scabra
Source: Sci Data. 2024 May 9;11:474. doi: 10.1038/s41597-024-03340-x (PMC11082199; doi:10.1038/s41597-024-03340-x)
Supplement: Supplementary file 1 — Supplementary table 1. [file 41597_2024_3340_MOESM1_ESM.docx]

**Supplementary table 1.** The sources of genome data for related species used in the phylogenetic analysis.

| Species name | Genomic data source |
| --- | --- |
| *Acanthaste Planci* | GCA_001949145.1 |
| *Lytechinus Variegatus* | GCA_018143015.1 |
| *Hemicentrotus Pulcherrimus* | GCA_003118195.1 |
| *Strongylocentrotus Purpuratus* | GCA_000002235.4 |
| *Pisaster Ochraceus* | GCA_010994315.2 |
| *Holothuria Leucospilota* | GCA_029531755.1 |
| *Plazaster Borealis* | GCA_021014325.1 |
| *Holothuria Glaberrima* | GCA_009936505.2 |
| *Patiria Miniata* | GCA_015706575.1 |
| *Anneissia Japonica* | GCA_011630105.1 |
| *Asterias Rubens* | GCA_902459465.3 |
| *Chiridota Heheva* | GCA_020152595.1 |
| *Apostichopus Japonicus* | GCA_037975245.1 |
| *Lytechinus Pictus* | GCA_037042905.1 |
| *Saccoglossus Kowalevskii* | GCA_000003605.1 |
| *Stichopus Monotuberculatus* | 10.6084/m9.figshare.22177898.v1 |
